# FedExosomes: Engineering Therapeutic Biological Nanoparticles that Truly Deliver

**DOI:** 10.3390/ph6050659

**Published:** 2013-04-29

**Authors:** Michelle E. Marcus, Joshua N. Leonard

**Affiliations:** 1 Interdepartmental Biological Sciences Graduate Program, Northwestern University Evanston, IL 60208-3120, USA; E-Mail: memarcus@u.northwestern.edu; 2 Department of Chemical and Biological Engineering, Chemistry of Life Processes Institute, Member, Robert H. Lurie Comprehensive Cancer Center, Northwestern University Evanston, IL 60208-3120, USA

**Keywords:** exosome, nanoparticle, siRNA delivery, extracellular vesicle, gene therapy, cancer, immunity

## Abstract

Many aspects of intercellular communication are mediated through “sending” and “receiving” packets of information via the secretion and subsequent receptor-mediated detection of biomolecular species including cytokines, chemokines, and even metabolites. Recent evidence has now established a new modality of intercellular communication through which biomolecular species are exchanged between cells via extracellular lipid vesicles. A particularly important class of extracellular vesicles is exosomes, which is a term generally applied to biological nanovesicles ~30–200 nm in diameter. Exosomes form through invagination of endosomes to encapsulate cytoplasmic contents, and upon fusion of these multivesicular endosomes to the cell surface, exosomes are released to the extracellular space and transport mRNA, microRNA (miRNA) and proteins between cells. Importantly, exosome-mediated delivery of such cargo molecules results in functional modulation of the recipient cell, and such modulation is sufficiently potent to modulate disease processes *in vivo*. It is possible that such functional delivery of biomolecules indicates that exosomes utilize native mechanisms (e.g., for internalization and trafficking) that may be harnessed by using exosomes to deliver exogenous RNA for therapeutic applications. A complementary perspective is that understanding the mechanisms of exosome-mediated transport may provide opportunities for “reverse engineering” such mechanisms to improve the performance of synthetic delivery vehicles. In this review, we summarize recent progress in harnessing exosomes for therapeutic RNA delivery, discuss the potential for engineering exosomes to overcome delivery challenges and establish robust technology platforms, and describe both potential challenges and advantages of utilizing exosomes as RNA delivery vehicles.

## 1. Introduction

Secreted extracellular vesicles are emerging as important new features of the expanding landscape of intercellular communication. Extracellular vesicles were first observed by Trams *et al*. in 1981 as particles that were shed from neoplastic cell lines and carried membrane-bound enzymes [1]. The authors noted that secreted extracellular vesicles could be taken up by recipient cells and presciently predicted that extracellular vesicles represented a physiological method for transferring information between cells, likening extracellular vesicles to liposomes used to package and deliver therapeutic molecules. A subset of extracellular vesicles in the 30–200 nanometer diameter range, known as exosomes, were subsequently found to play a number of important roles in intercellular signaling, including shedding of obsolete proteins during reticulocyte maturation [2], presentation of antigens to T cells [3], activation of B and T cell proliferation [4], and induction of immune rejection of murine tumors, presumably by delivery or presentation of tumor antigens to the immune system [5]. The distinctions between exosomes and other extracellular vesicles (such as microvesicles, which bud from the plasma membrane) is a topic of some nuance and controversy, which is beyond scope of this review but is discussed in detail elsewhere [6,7]. Here, we focus our discussion on exosomes, noting the caveat that few published investigations in this field have explicitly distinguished between exosomes and related extracellular vesicles. Exosomes have generated great interest for their roles in intercellular communication and their potential to therapeutically modulate immune cell signaling. Subsequent investigations into exosome biogenesis, cargo packaging, and mediation of intercellular communication have identified new opportunities for harnessing and modifying exosomes to develop exosome-based therapeutics.

### 1.1. Exosome Biogenesis

**Figure 1:**
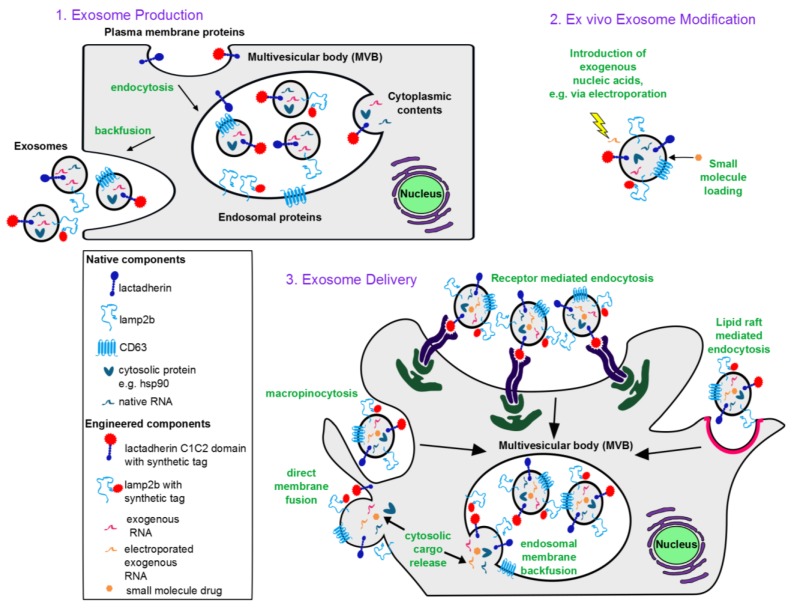
Conceptual overview of exosome-based therapeutics: (1) *Exosome biogenesis*. Exosomes incorporate membrane components from the plasma and endosomal membranes, cytoplasmic proteins and RNA. Plasma membrane proteins reach exosomes via endocytosis into the endosomes followed by invagination of the endosomal membrane to form intraluminal vesicles (intracellular precursors of exosomes). An endosome containing many such intraluminal vesicles is termed a multivesicular body. Upon invagination of the endosomal membrane, endosomal membrane proteins also get incorporated into intraluminal vesicles. During invagination, cytoplasmic contents including RNA and proteins are engulfed into the lumen of the intraluminal vesicles. Upon backfusion of the multivesicular body with the plasma membrane, intraluminal vesicles are released into the extracellular space and are then termed exosomes. (2) *Ex vivo modification of exosomes*. Nucleic acids can be introduced to the exosome lumen via electroporation, and lipophilic small molecules can be passively loaded. (3) *Exosome delivery*. Exosomes are internalized by recipient cells via macropinocytosis, receptor-mediated endocytosis, or lipid raft-mediated endocytosis, each of which results in exosomes being taken up into endosomes. Exosomal contents are then released into the cytoplasm via backfusion with the endosomal membrane. Alternatively, exosomes can fuse directly with the recipient cell plasma membrane to release exosomal contents into the cytoplasm. Mechanisms of internalization utilized depend on the ligands displayed on the exosome surface, the cell type from which the exosomes are derived, and the recipient cell type.

Exosomes have been discovered in the supernatants of a wide variety of cells in culture, and are present in all human bodily fluids, suggesting that they can be produced by any type of cell [8]. Exosomes are the extracellular equivalent of intraluminal vesicles (ILVs). ILVs are formed when the limiting membrane of an endosome buds inward, forming an internal vesicle (Figure 1). Endosomes containing ILVs are known as multivesicular endosomes or multivesicular bodies (MVBs). Although some MVBs traffic along the endosomal pathway towards the lysosome, other MVBs back fuse with the plasma membrane, releasing their contents, including ILVs, into the extracellular space. ILVs that have been released into the extracellular space are known as exosomes. Exosomes are therefore topologically equivalent to cells, encapsulating cellular cytoplasmic contents in the exosomal lumen and presenting membrane protein domains on the exosomal exterior that correspond to domains presented at the cell surface and in the lumen of the endoplasmic reticulum [9].

### 1.2. Characteristic Exosome Contents

Exosomes are enriched in particular cellular proteins, including the tetraspanins CD63, CD9, and CD81, ESCRT related proteins Alix and Tsg101, MHCI, and heat shock proteins [8]. Exosomes derived from immune cells are also enriched in MHCII and costimulatory molecules [10]. Exosomes also package cellular RNAs and protect them from degradation [11], such that exosomes isolated from serum contain RNA that represents a subset of the RNA present in the exosome-producing cells [12]. Thus, exosomes and their contents are potentially very useful as biomarkers, particularly for diseases such as cancer wherein the diseased cells produce many exosomes. For these reasons, diagnostic analysis of exosomal proteins and RNA has received much attention and has been commercialized (reviewed in [8]). Furthermore, exosomal proteins and RNA play functional roles in exosome-mediated intercellular communication. Exosomal mRNA is expressed in recipient cells [11], and exosomal microRNA (miRNA) inhibits gene expression in recipient cells [13]. Exosomal proteins play a role in adhesion to and uptake by recipient cells [14], participate in transcriptional regulation [15], and bind recipient cell receptors to modulate signaling pathways [16,17].

Though less intensively studied, other exosomal components also play important roles in exosome-mediated signaling. Exosomal lipid composition is distinct from that of the outer cell membrane; exosomes are enriched in sphingomyelins, phosphatidylserine, and cholesterol [8]. It has been hypothesized that this unique lipid composition facilitates uptake of exosomes by recipient cells [14]. Exosomes also display polysaccharides and are enriched in complex N-linked glycans [8]. In addition to these commonly reported exosome components, a wide variety of biomolecules have been reported in exosomes, to such an extent that Mathivanan *et al*. have developed ExoCarta, an online compendium of proteins, mRNAs, miRNAs, and lipids that have been documented in exosomes [18]. The mechanisms by which various components are targeted for incorporation into exosomes are poorly understood and appear to differ depending on context and cell of origin. Nonetheless, the observations that exosomes play an important role in intercellular signaling and that the composition of exosomes is distinct from that of their cells of origin suggest that packaging of molecules into exosomes is, to some extent, a regulated process, which we discuss below when considering specific types of exosomal cargo.

Some exosomal contents play a role in disease processes when exosome-mediated transfer is hijacked by viruses and tumors to increase viral spread or cancer growth and metastasis, respectively [19]. For example, exosomes derived from nasopharyngeal cancer cells deliver miRNAs and proteins to endothelial cells and upregulate the growth of these cells, inducing angiogenesis and supporting tumor survival [19]. Tumor-derived exosomes are also increasingly recognized as major players in shaping the tumor microenvironment and immune response, generally promoting tumor survival (reviewed previously, [10,20]). Epstein Barr virus (EBV)-infected B cells transfer EBV miRNAs to dendritic cells (DC) and down-regulate the anti-EBV immune response [21]. Whether such opportunistic coopting of exosomal transfer involves mechanisms for targeted packaging of specific cargo molecules remains to be elucidated. Overall, these diverse exosomal contents induce distinct functional consequences in recipient cells (as summarized in Table 1), and these effects present both opportunities and challenges for translating exosomes to the clinic.

**Table 1:** Functional consequences of exosome delivery to recipient cells.

Exosome source	Recipient cell type	Cargo delivered	Functional consequences	Ref.
*Immunosuppressive effects*				
EBV transformed human B cells	Human Monocyte-derived DC	Viral miRNA	Down-regulate immune response to virus	[21]
Serum of pregnant human patients	Human Jurkat T cells	FasL	Suppress CD3ζ signaling and IL-2 production	[22]
Murine BMDC overexpressing IL-10	Murine T cells	Antigen, presented on MHCII	Suppress T cell proliferation	[23]
*Immunostimulatory effects*				
Murine BMDC	Murine CD8^+^ and CD4^+^ T cells (*in vitro* and *in vivo*)	Antigen, presented on MHC	Induce T cell proliferation	[24,25]
CD28 stimulated human CD3^+^ T cells	Unstimulated human CD3^+^ T cells	Unidentified	T cell activation, induction of proliferation and cytokine production when co-delivered with IL-2	[26]
Murine BMDC	Murine BMDC (allogeneic)	Antigen	Transfer of foreign antigen, followed by foreign antigen presentation to and activation of T cells	[14]
*Therapeutic effects*				
Human H9 CD4^+^ T cells	Human Jurkat T cells, Human PMBC	APOBEC3 protein (HIV replication inhibitor)	Reduce HIV replication	[27]
Human Endothelial cells	Human Aortic Smooth Muscle Cells	miR-143, miR-145	Reduce atherosclerotic lesions	[28]
Murine MSC	Murine Primary Neurons	miR-133b	Neurite outgrowth after injury	[29]
*Pathogenic effects*				
Human B cell lymphoma cell lines	None		Bind and sequester rituximab (antibody used in B cell lymphoma immunotherapy)	[30]
Human CSF	None	Phosphorylated tau	Transport of neurotoxic protein in Alzheimer’s disease	[31]
Human PMBC derived DC incubated with HIV	Jurkat T cell line expressing CCR5	HIV viral particles	Delivery of functional HIV viral particles encapsulated in exosomes, leading to HIV infection of recipient cells	[32]

## 2. Opportunities and Challenges in Harnessing Exosomes for Therapeutic Applications

Because exosomes are produced naturally by most cells in the body and natively transport biological information between cells, it is possible that exosomes are well-suited to delivery of therapeutic molecules as well. Here we highlight the opportunities and challenges associated with harnessing exosomes for therapeutic applications.

### 2.1. Therapeutically Attractive Exosome Properties

#### 2.1.1. Intrinsic Therapeutic Activity

For some applications, unmodified exosomes natively exhibit desirable therapeutic activities. A well-studied application is using exosomes derived from DC, which include peptide-MHC complexes that can be transferred to recipient cells, for vaccination. For example, intradermal delivery of exosomes derived from DC pulsed with tumor peptide induced an immune resonse that inhibited mastocytoma tumor growth in mice [5]. Exosomes from DC pulsed with diphtheria toxin (DT) induced DT-specific antibody production when administered intravenously (i.v.) to mice [33]. Similarly, i.v. injection of exosomes derived from DC pulsed with *Leishmania major* antigens protected mice from *L. major* infection [34]. Mesenchymal stem cell (MSC)-derived exosomes also exhibit therapeutic properties in a variety of contexts. MSC-derived exosomes administered to mice with myocardial ischemia/reperfusion injury reduced myocardial infarct size relative to the area at risk for infarct [35]. MSC-derived exosomes also induced neurite growth in rat primary neurons after middle cerebral artery occlusion, indicating that these exosomes may have neuroprotective effects [29]. Furthermore, MSC-derived exosomes can inhibit hypoxia-induced pulmonary hypertension in mice [36]. Adipose-derived MSC exosomes contain neprilysin, an enzyme that degrades the pathogenic β-amyloid peptide, and can decrease β-amyloid levels in neural cells [37]. Exosomes derived from other cell types also exhibit therapeutic properties. Human natural killer (NK) cell-derived exosomes, when incubated with tumor cell lines, promote tumor cell lysis and may play a role in inhibiting tumor growth *in vivo* [38]. Exosomes can also transfer antiviral protein APOBEC3G between T cells, conferring HIV protection to recipient T cells [27]. Endothelial cell-derived exosomes deliver miR-143 to aortic smooth muscle cells, which can reduce atherosclerotic lesions in mice fed a high-fat diet [28]. These therapeutic applications of unmodified exosomes indicate that exosome-mediated therapy is potentially safe and that exosome-mediated delivery is sufficiently efficient to confer therapeutic benefits.

#### 2.1.2. Immunological Compatibility

A key potential benefit of using exosomes therapeutically is their potential to mediate gene delivery without inducing adverse immune reactions. In contrast, many commonly used gene therapy vehicles including viral vectors and lipid nanoparticles activate the host immune system. Such immune activation limits the repeat administration of the gene therapy vector, and in some cases, necessitates the co-administration of immunosuppressive drugs [39]. By comparison, repeated i.v. administration of autologous exosomes derived from immature DC did not stimulate anti-exosome immune responses in mice [40]. There is some evidence that allogeneic exosomes are also tolerated *in vivo*. For example, when exosomes derived from BALB/c DC were injected i.v. into B10 mice, splenic DC subsequently isolated from the recipient mice did not display maturation markers or enhanced capacity to stimulate T cell proliferation. However, treatment with such allogeneic exosomes did not block activation of splenic DC *in vivo* using an antagonistic anti-CD40 antibody, suggesting that allogeneic exosomes were neither profoundly immunostimulatory nor entirely immunosuppressive, at least by the measures considered in this investigation [14]. To some extent, immune tolerance appears to even extend between species. For example, exosomes derived from human MSC were tolerated and functional in immune-competent mice [35], and exosomes derived from human HEK293 cells were tolerated and functional in T cell deficient (RAG2^−/−^) mice [41]. However, neither of these investigations described repeated administration of such exosomes. Whether allogeneic exosomes are tolerated in humans has yet to be established, and such investigations would need to consider risks of acute inflammation, induction of autoimmune complications, and perhaps even transfer of pathogens including endogenous retroviruses [42].

Although most exosomes appear to escape immune surveillance, some exosomes may also *actively* suppress immune activation. For example, exosomes derived from the placenta are well known suppressors of the maternal immune response to the fetus. Placental exosomes display FasL and inhibit T cell activation by suppressing CD3ζ signaling and IL-2 production [22]. Exosomes produced by immune cells can also be immunosuppressive. Activation-induced T cell death is partially mediated by FasL-expressing exosomes, which are released from activated T cells [43]. Activated OVA-specific CD8^+^ T cells produce exosomes that inhibit OVA antigen presentation by DC, resulting in decreased anti-OVA CTL responses [44]. Furthermore, administration of exosomes from donor immature DC prior to heart transplant administration decreases graft rejection in mice and increases the fraction of splenic T cells expressing FOXP3 (a marker of regulatory T cells) [45]. In addition, the tolerogenic properties of DC-derived exosomes can be enhanced by engineering the exosome-producing DC. DC treated with recombinant IL-10 and transduced DC overexpressing IL-10, IL-4, or FasL generated exosomes capable of reducing inflammation in mouse DTH and collagen induced arthritis models [23,46,47].

In practice, exosome source and mechanisms of immunological compatibility must be paired with the requirements of the target application. For example, exosomes from immature DC are more likely to produce a general tolerogenic response than are activated T cell-derived exosomes, which induce antigen-specific tolerance. Finally, it would be feasible to generate autologous DC-derived exosomes, which is far more practical and broadly applicable than generating placenta-derived exosomes. It may even be possible to design or engineer cell-based therapies that continuously produce exosomes *in vivo* to obviate the need for repeated exosome injections and thus control adverse inflammatory responses and provide therapeutic benefits in a sustained fashion.

#### 2.1.3. Cargo Versatility

Exosomes are well-suited to delivering a variety of biologically active cargos. Exosomes naturally deliver mRNA, miRNA, various noncoding RNA, mitochondrial DNA, genomic DNA, and proteins [11,39,48,49]. However, the efficiency of exosome-mediated delivery has not yet been systemically evaluated, and it is likely to vary based on recipient cell type, exosome source, and cargo of interest. In particular, there are likely to be differences in the ability of exosomes to package and deliver cargos of various sizes and molecular structures, since it is now well-documented that exosomes naturally package a subset of cellular RNAs and proteins that is related to but not identical to that found in the exosome-producing cell [11,50]. Developing exosomes as delivery vehicles will require both characterizing the delivery efficiency of specific cargos to specific cell types and systematically comparing this efficiency with that mediated by existing state-of-the art delivery vehicles.

#### 2.1.4. Experience in Clinical Trials

Exosomes have already been approved for use in clinical trials, and our experience with exosome-based therapies in humans is rapidly expanding [51]. In one trial, DC from patients with advanced metastatic melanoma were loaded with melanoma antigen *ex vivo*. Exosomes from these DC were then isolated and administered in an autologous fashion in an attempt to promote anti-melanoma immunity via therapeutic vaccination. In some patients, minor inflammatory responses at the site of exosome injection (mild swelling, redness, DTH responses) and low-grade fever were observed after exosome administration. However, patients tolerated repeated administration of autologous exosomes for up to 21 months [52]. In a similar trial, non-small-cell-carcinoma lung cancer patients were injected with autologous exosomes weekly for 4 weeks, and similar low level immune responses were observed [53]. Finally, in a clinical trial in which tumor ascites-derived exosomes were isolated and reintroduced along with granulocyte macrophage colony-stimulating factor (GM-CSF), the only adverse response to exosome vaccination reported was mild inflammatory responses at the site of vaccination [54].

The main conclusion to be drawn from clinical trials to date is that repeated administration of autologous exosomes is well-tolerated in humans. Although therapeutic benefits following exosome vaccination have not yet been proven, several patients in these phase I clinical trials exhibited a halt in disease progression after exosome vaccination [53,54]. These trials thus also suggest that exosome administration is sufficient to generate some physiological responses. Together, these observations support the argument that exosome-based therapeutic delivery vehicles may also be well-tolerated and efficacious in humans.

### 2.2. Challenges for Realizing Exosome-Mediated Therapeutics

#### 2.2.1. Exosome Isolation and Purification

Multiple methods have been described for purifying exosomes, but each presents unique challenges for clinical translation. For a summary of these methods, see Table 2. Exosomes can be isolated from conditioned cell culture media or bodily fluids by differential centrifugation, filtration paired with centrifugation, high-performance liquid chromatography paired with centrifugation, adsorption to antibody-coated beads, or polymer-based precipitation [55,56]. Each of these methods is time-consuming and labor intensive, requiring hours to isolate exosomes from conditioned media. Bead and polymer-based isolation methods generally require overnight incubation steps, further increasing the time required to isolate exosomes. Furthermore, for best yield, exosomes must be isolated from the conditioned media of cells cultured for 3–6 days. Even under optimized conditions, exosome isolation methods can yield extremely low levels of exosomes. High-performance liquid chromatography is a more scalable technology, but methods described to date generate low yields of exosomes [56]. Because bead-based isolation of exosomes depends on antibody recognition of exosomal proteins, only a subset of all exosomes (those expressing the antibody-recognized protein) can be captured. While differential centrifugation has the potential for higher exosome yields, this method is subject to operator-dependent variability [57]. Polymer precipitation-based purification may reliably produce high yields of exosomes [56], but residual polymer carrier remains in these preparations.

**Table 2:** Exosome isolation methods

Isolation method	Advantages	Disadvantages	Ref.
Differential centrifugation	Potentially high yields Potentially sterile	Time-consuming Subject to operator-based variability	[55,57]
HPLC + centrifugation	High throughput	Low yields	[56]
Affinity beads	High throughput Fewer steps than centrifugation methods	Selection of exosome population subset Difficulty in completely removing antibody from sample	[57]
Polymer-based precipitation	Potentially high yields Fewer steps than centrifugation methods	No method for removing polymer from exosome sample	[56]
Filtration + centrifugation	Potentially high yields Sterile	Time-consuming Subject to operator-based variability	[57]

For translation of exosomes to the clinic, the exosome preparations must be pure and sterile, which presents challenges specific to each purification method [57]. For example, there is currently no method for removal of polymer from exosome samples isolated by polymer-based precipitation methods, and the effects of such polymer contamination have not yet been studied *in vivo*. In centrifugation-based methods, the need for multiple centrifugation steps increases the risk of sample contamination. However, filtration combined with centrifugation has been successfully used to develop sterile clinical grade exosomes [57]. Elution of exosomes from antibody-coated beads risks contamination by bead-derived antibodies. Finally, exosomes are heterogenous in size and protein composition [55], and therefore any method of exosome production must be rigorously characterized to determine the degree of variability within and between batches. It is possible that some of these challenges may be addressed using ever-advancing technologies and experience developed in the related fields of cell-based therapies and cell-derived biologics. In any application, the production and purification challenges will be highly dependent on the cellular source of the exosomes.

#### 2.2.2. Selecting and Culturing Exosome-Producing Cells

Exosome contents are strongly influenced by the producer cells from which the exosomes are derived, and exosomes derived from different cells exert vastly different functional effects on recipient cells (Table 1). Moreover, even exosomes derived from a single source can exhibit a multitude of effects on recipient cells. For example, murine cardiomyocyte-derived exosomes induced significant changes in the expression of 161 genes in murine fibroblast recipient cells [49]. Therefore, developing safe and effective exosome-based therapeutics will require both careful choice of exosome producer cells and analysis of exosome contents and their biological effects on recipient cells.

Most clinical strategies investigated to date involve the use of autologous exosomes, and therefore producer cell types considered for clinical applications are typically those with which there exists extensive experience in the field of autologous cell-based therapies. For example, one potential source of therapeutic exosomes are immature DC, which may be derived from CD34+ cells isolated from a patient’s peripheral blood. Similarly, MSCs can be derived from a patient’s bone marrow, fat, or other tissue. MSC-derived exosomes are attractive because they mediate immunosuppressive effects of MSC-based therapies [58].

Exosome contents and functional effects are likely to depend on both the type of producer cell used and the way in which these cells were cultured. For example, exosomes derived from immature DC do not induce immune activation [40], whereas exosomes derived from mature DC are immunostimulatory and can prime vaccination [24]. To some extent, clinical experience with cell-based therapies provides insights into the safety and potential side-effects of exosome-based therapies. For example, DC-derived exosomes are already introduced into humans as part of any DC-based immunotherapy. In some cases, this experience warrants caution, for example noting that MSC-derived exosomes have been implicated in promoting tumor vascularization [59].

Whether exosomes must be derived from autologous cells for all applications remains to be determined, since some evidence from animal models suggests that exosomes from other species may be tolerated to some extent [35,40]. Ultimately, it may be possible to engineer “universal donor” exosome-producing cell lines, which would drastically reduce the cost and complexity of producing exosome-based therapies. For some applications, it may also prove necessary to develop strategies that enrich exosomes for desirable components and remove or prevent the incorporation of problematic or hazardous components that confer undesirable effects on recipient cells. Thus, optimizing the safety, efficacy, and cost-effectiveness of exosome-based therapies will require careful selection of exosome-producing cells and potentially modification of the exosomes or the cells from which they are produced.

## 3. Engineering Exosomes as Therapeutic Delivery Vehicles

### 3.1. Incorporating Therapeutic Molecular Cargo into Exosomes

Therapeutically active exosomal cargo is not limited to the native producer cell-derived biomolecules discussed above. Rather, exosomes may also be loaded with exogenous molecules conferring desirable therapeutic activity. To date, exosomes have been engineered to incorporate a variety of therapeutic molecules, including protein and peptide ligands [40,60], small molecule drugs [61], and therapeutic RNA [40,62]. Below, we discuss both methods and applications for incorporating exogenous therapeutic biomolecules into exosomes.

#### 3.1.1. Protein Cargo

The most common method for engineering the incorporation of a “cargo” protein or peptide into exosomes is via genetic fusion of the cargo-encoding gene to the gene encoding a protein known to localize to exosomes. N-terminal fusion of an OVA peptide antigen to the C1C2 domain of lactadherin (an abundant exosomal protein) achieved display of the OVA peptide on the exterior of exosomes [63]. This mechanism was also used to display the protein antigen, HER2 [64], demonstrating that both proteins and peptides can be targeted to the surface of exosomes via fusion to lactadherin C1C2. Other fusion partners include N-terminal fusion of the FLAG peptide to the lysosomal protein Lamp2, which achieved display of this peptide on the exosomal exterior [40]. In yet another example, fusion of the GE11 peptide to the transmembrane domain of platelet-derived growth factor receptor generated GE11^+^ exosomes [41]. Notably, each of these strategies resulted in display of engineered proteins on the exosome surface, but fusion of exogenous peptides to proteins that localize to the exosome lumen (such as heat shock proteins [8]), has not yet been investigated.

An alternative method of targeting proteins to exosomes derives from the observation that in general, oligomeric membrane-anchored proteins traffic to exosomes [60]. The authors suggest that this occurs due to the targeting of such proteins to sites of vesicle budding at the plasma membrane. Thus, proteins can be targeted to exosomes via fusion of the cargo protein to an aggregation-mediating domain, conferring oligomerization of the cargo protein, and a protein sequence to which a myristoyl moiety is added, conferring membrane localization of the protein. This strategy was used to target GFP to exosomes [60], but this approach has not yet been applied to incorporation of functional proteins into exosomes. Indeed, the requirement of generating protein oligomers would be undesirable in many applications, in which the cargo protein is intended to play a functional role in the recipient cell. On the other hand, while this study did not investigate the topology of the targeted protein in the exosome, this strategy likely localizes proteins to the exosome lumen because these proteins are associated with the cytoplasmic face of the plasma membrane. This targeting strategy provides a mechanism by which protein cargo could interact with other luminal cargo, including nucleic acids.

#### 3.1.2. RNA Cargo

One method of introducing exogenous RNA into exosomes is via electroporation of purified exosomes. Alvarez-Erviti *et al*. pioneered this method, electroporating siRNA into DC-derived exosomes and achieving up to 60% RNA and protein knockdown of GAPDH in the mouse midbrain, cortex, and striatum upon i.v. delivery of electroporated exosomes. The same strategy achieved similar knockdown efficiency with BACE-1 siRNA, suggesting that the method is independent of siRNA sequence [40]. In another example, exosomes derived from peripheral blood mononuclear cells from human plasma were electroporated with siRNA against MAPK-1. These electroporated exosomes knocked down MAPK-1 expression in lymphocytes and monocytes from healthy human donors [65]. Electroporation of siRNA into exosomes derived from HEK 293 cells has also been reported [66]. Together, these results suggest that this strategy is broadly applicable to a variety of exosome sources and types of recipient cells. However, electroporation may not be effective for all types of RNA cargo. For example, miRNA, shRNA, mRNA, or RNAs containing chemical modifications have not yet been electroporated into exosomes. In fact, Ohno *et al*. reported that they were unable to electroporate miRNA into HEK 293-derived exosomes, suggesting that some sizes or conformations of RNA may be less amenable to this approach [41].

A less targeted but commonly utilized strategy for incorporating RNA into exosomes comprises simply overexpressing the cargo RNA in the exosome-producing cells. This method potentially utilizes a mass action driving force to promote nonspecific incorporation of cargo RNA into exosomes. Such cargo RNA overexpression in producer cells has been used to incorporate miRNA [41,67,68], chemically modified 3' benzen-pyridine miRNA [62], shRNA [67], and mRNA [28,69] into exosomes. Upon incubation of exosomes carrying these RNAs with recipient cells, these overexpressed RNAs were all functional; the mRNA was translated into protein, and the shRNAs and miRNAs induced target gene knockdown. This strategy thus appears to be broadly applicable to a variety of RNA cargos and recipient cell types. Interestingly, overexpression of mRNA in exosome-producing cells also results in high expression of the protein for which this mRNA codes, and this protein is packaged into exosomes as well [69]. However, this mass action-driven strategy is not typically employed to intentionally incorporate cargo proteins into exosomes, since there exist more efficient targeted methods for achieving protein incorporation, as discussed above.

A new and exciting frontier is the identification and utilization of native mechanisms for packaging specific RNA molecules into exosomes. One such strategy is the use of RNA zipcodes, which are sequence motifs in the 3' untranslated region (UTR) that direct mRNA localization within the cell. Bolukbasi *et al*. identified two features—a miR-1289 binding site and a core “CTGCC” motif—that are enriched in the 3' UTRs of a large proportion of mRNAs found in glioblastoma- and melanoma-derived exosomes. Replacing the 3' UTR of eGFP with a 25 nucleotide sequence containing the miR-1289 binding site and the “CTGCC” motif added was sufficient to increase eGFP mRNA incorporation into HEK293T exosomes by 2-fold compared to untagged eGFP mRNA. Overexpression of miR-1289 further increased the incorporation of the construct 6-fold compared to the untagged eGFP mRNA. This increase in exosome targeting depended on the presence of the miR-1289 binding site, as mutation of this site abrogated enrichment of the mRNA in exosomes [70]. As our understanding of native mechanisms by which RNA is packaged into exosomes develops, so may our toolbox for engineering the incorporation of specific cargo RNAs into exosomes.

### 3.2. Targeting Exosome Delivery

#### 3.2.1. Targeting Exosomes to Specific Recipient Cells

Developing safe and effective exosome-based therapeutics requires assessing and potentially modulating the cells and subcellular compartments to which exosomes are targeted. Although the pharmacokinetics of systemically administered exosomes has not been characterized in detail, i.v. administration of purified exosomes to mice resulted in accumulation of exosomes in the liver, kidney, and spleen [40,41]. This biodistribution profile is consistent with that of most nanoparticle delivery vehicles, which are generally cleared from circulation through biliary excretion, renal clearance, or uptake by macrophages in the reticuloendothelial system [71]. However, this biodistribution may be altered by targeting exosomes to specific cellular receptors, and several strategies for achieving such targeting have been reported.

One strategy for targeting exosomes to specific cell types is to harness virus-derived proteins and peptides that have evolved precisely to confer such targeted delivery. For instance, exosomes engineered to display EBV glycoprotein 350 target and deliver protein antigens to CD19^+^ B cells but not to other peripheral blood mononuclear cells (PBMC) [72]. By conferring cell-type specificity, viral ligands can increase exosome-mediated cargo delivery to target cells. Exosomes displaying a central nervous system-specific rabies viral glycoprotein (RVG) peptide readily delivered siRNA to neural cells, whereas unlabeled exosomes did not. Delivery of siRNA by RVG-tagged exosomes to neural cells was dependent on the ability of the RVG peptide to bind the acetylcholine receptor [40]. This strategy was also effective *in vivo*, where RVG tagged exosomes readily delivered siRNA to the mouse brain after i.v. injection, whereas untagged exosomes delivered siRNA to the mouse spleen, liver, and kidney [40]. Repeat doses of RVG-tagged exosomes did not induce inflammation. In addition to enhancing exosome targeting, viral components can also enhance release of exosomal cargo in recipient cells. Display of the viral protein VSV-G on exosomes was used to increase the potency of an exosomal vaccine. Exosomes tagged with VSV-G and OVA peptide were taken up by DC at an enhanced rate compared to exosomes tagged with OVA alone. A fusion-deficient mutant of VSV-G did not confer similar enhancement of vaccination [73]. Despite the success of using viral components for exosome targeting, this strategy is limited to known interactions between viral proteins and cellular receptors. Furthermore, although repeat doses of RVG-tagged exosomes did not induce inflammation in mice [40], the potential for viral components to promote an immune response against therapeutic exosomes remains unclear. Therefore, the use of viral components for targeting exosomes must be carefully evaluated for undesirable side effects, and this analysis is likely to be application-specific.

An alternative targeting approach is to utilize engineered peptide ligands. Although this strategy has not yet been applied to exosomes, the use of antibody fragments specific for epitopes displayed on target cells is a common strategy for targeting nanoparticle drugs and is currently being evaluated in a variety of clinical trials [74]. Another method of generating engineered targeting ligands is phage display. Using this approach, exosomes displaying the GE11 peptide, which targets epidermal growth factor (EGFR), accumulated in EGFR^+^ human xenograft tumor models in mice to levels three times higher than those observed using non-targeted exosomes [41]. However, in this study, GE11-tagged and non-targeted exosomes both accumulated in the liver to a similar extent, as measured by *in vivo* imaging of PKH67 labeled exosomes. This suggests that not all exosome targeting methods can mitigate off-target exosome accumulation. Furthermore, not all ligand-receptor interactions confer exosome targeting *in vivo*. For example, a muscle-specific peptide identified by phage display enhanced exosome-mediated delivery of siRNA to muscle cells *in vitro* but did not confer similar effects *in vivo* after i.v. injection [40]. In general, the overall targeting efficiency conferred by each ligand-receptor pair is characterized by multiple properties, including ligand-receptor affinity, induction of downstream signaling pathways, and utilization of specific modes of exosomal uptake and intracellular trafficking. Therefore, an optimal exosome-mediated delivery strategy should target both desired cell types as well as intracellular mechanisms that best confer delivery of cargo molecules.

#### 3.2.2. Targeting Exosome-Mediated Delivery to Specific Subcellular Locations

The mechanism by which exosomes are taken up by recipient cells is not fully understood, although most evidence suggests that exosomes are usually taken up into endosomal compartments via endocytosis [13,14,75], macropinocytosis [75,76], or phagocytosis [13,74,75]. Moreover, both direct and indirect evidence indicate that exosomal contents are delivered to the cytoplasm of the recipient cell, which is typically desirable for delivery of therapeutic RNA. For example, DC expressing luciferase were treated with exosomes loaded with the luciferin substrate, and generation of bioluminescence confirmed mixing of exosomal contents and cytoplasmic compartment [13]. This content mixing could result from either direct fusion of exosomes with the DC plasma membrane or from back-fusion of endocytosed exosomes with the endosomal membrane. Either of these mechanisms would deliver cargo RNA to the cytoplasm, although because RNA-induced silencing complex (RISC), miRNA, and miRNA-repressible mRNA associate with MVBs [77,78], delivery via exosomal back-fusion might lead to greater RNAi efficiency due to the proximity of these downstream molecules. Moreover, the fact that siRNA delivered by exosomes can induce RNAi-mediated knockdown of target gene expression in recipient cells also indicates that at least some percentage of exosomal content reaches the recipient cell cytoplasm. The mechanism by which exosomes deliver RNA to the recipient cell cytoplasm is not well understood and may vary among different types of exosomes and recipient cells. In addition, some exosomes may also reach the recipient cell nucleus [49], although whether such trafficking is mediated by a specific cellular mechanism is unknown.

Interactions between ligands naturally displayed on exosomes and receptors on recipient cells play an important role in exosome uptake by the recipient cell. Morelli *et al*. found that antibody-mediated blockade of adhesion molecules CD11a, CD54, or tetraspanins CD81 or CD9 on the surface of DC-derived exosomes, or simultaneous blockade of α_v_ and β_3_ integrins on recipient BMDC, significantly diminished DC-derived exosome uptake by recipient BMDC. Furthermore, exosome uptake was decreased in the presence of competitive RGD hexapeptide [14]. However, the involvement of a given ligand in exosome uptake appears to be context-specific, as competitive RGD peptide was found not to inhibit the uptake of oligodendrocyte-derived exosomes by microglia [76].

The involvement of peptide ligands in exosome uptake suggests that clathrin-mediated endocytosis may be a mechanism of exosome uptake. In support of this hypothesis, Escrevante *et al*. observed that inhibition of clathrin-mediated endocytosis with chlorpromazine decreased uptake of SKOV3 ovarian cancer-derived exosomes by SKOV3 recipient cells [75]. However, this study also found that blocking other modes of internalization, including phagocytosis and macropinocytosis, also reduced exosome uptake, suggesting that exosomes may be internalized via multiple mechanisms, even within a single cell.

Despite the important role played by proteins displayed on the surface of exosomes, such proteins may not always be required for exosome uptake. Treatment of SKOV3 exosomes with proteinase K prior to incubation with SKOV3 recipient cells significantly decreased, but did not abolish, exosome uptake [75]. Exosomal glycan ligands may also play a role in uptake, as blocking of exosomal β-galactosides with galectin-5 decreased macrophage uptake of reticulocyte-derived exosomes [79]. Finally, interactions between exosomal and cellular lipids play a role in exosome uptake, since exosome uptake is decreased in the presence of a soluble phosphatidylserine analog that competes with exosomal phosphatidylserine for recognition by recipient cells [14]. Furthermore, when cells were pre-treated with the cholesterol-sequestering agent filipin, exosome uptake was reduced [13,80], suggesting that in some cases exosomes may be taken up by lipid-raft-mediated endocytosis.

Despite the many potential mechanisms of exosome uptake, most evidence suggests that the majority of exosomes are internalized via endocytic pathways. However, the efficiency with which exosomes escape the endosomal system and deliver cargo to the cytoplasm is unknown. The efficiency of this process can be increased, however, by functionalizing exosomes with cell-penetrating peptides, which are short cationic or amphipathic peptides capable of inducing fusion between cellular membranes [66]. While cell-penetrating peptides may induce direct fusion between exosomes and the recipient cell’s outer plasma membrane, it is usually observed that conjugation of cell-penetrating peptides to lipid particles causes uptake of the conjugate by endocytosis, followed by fusion of the lipid carrier with the endosomal membrane [81]. Fusion of cell-penetrating peptides to exosomes may enhance their escape the endosomal system and increase their delivery of cargo to the cytoplasm of recipient cells.

## 4. Reverse Engineering Exosomes: Designing Exosomal Features into Synthetic Vectors

Elucidating the mechanisms by which exosomes mediate intracellular delivery of biomolecules may also identify design strategies to guide the development of “synthetic exosomes.” For example, artificial lipid vesicles such as liposomes could provide a foundation for building synthetic exosomes. Liposome composition could be altered to match the lipid composition of exosomes, which may help to reduce adverse immunological events such as binding of complement proteins or production of IgM (against PEGylated liposomes) and increase liposome half-life in circulation [82]. Exosomes are enriched in sphingomyelins, cholesterol, glycolipid GM3, and glycerophospholipids with long and saturated fatty acyl chains. Rigid lipid compositions such as those of exosomes may increase circulation stability of lipid particles, which may be beneficial for increasing liposome half-life in the blood [83]. This lipid composition also decreases uptake of lipid vesicles by macrophages *in vitro* and thus may decrease RES clearance [84,85]. While decreasing liposome uptake by macrophages in the RES is desirable (unless macrophages are the target cell type), the liposome must be taken up efficiently by target cells. Some exosomal components, including phosphatidylserine, integrins, and tetraspanins, increase uptake by recipient cells [14]. Thus, when combined with cell-specific targeting ligands, mimicking exosomal features that enhance uptake by recipient cells may be useful for enhancing liposome-mediated delivery of cargo molecules to target cells.

Other exosomal features could be recapitulated to decrease liposome-mediated immune stimulation. For example, liposomes could be decorated with proteins that confer immunosuppressive properties to exosomes, such as FasL. Exosomes also display complement inhibitors CD55 and CD59 [86], which minimize exosome lysis upon incubation with serum. Since liposome-mediated activation of complement can induce lethal immune stimulation [87], incorporation of complement inhibitors found in exosomes may increase the safety of liposomes as gene delivery vehicles.

Finally, in the effort to elucidate physiological roles and biophysical properties of exosomal components, liposomes could be a valuable tool for displaying defined exosomal components, either individually or in combination. This would allow for a better understanding of the isolated effects of each component of exosomes, as well as providing a method for discovering potential synergistic effects between combinations of components. Such characterization, in turn, would enable one to incorporate desirable exosomal features into the design of better artificial gene delivery vehicles. This analysis could also inform the selection or design of ideal exosome producing cells, potentially including genetic modifications to exclude harmful components from exosomes. Thus, efforts to engineer exosomes and exosome-inspired synthetic vectors should dovetail to yield a greater understanding of both technologies and enable the development of improved gene delivery vectors.

## 5. Conclusions

Exosomes are newly appreciated but important mediators of intracellular communication, enabling a “producer” cell to directly alter the functional state of a “recipient” cell by delivering protein and nucleic acid cargo. Although the field of engineered exosomes is still in early stages, our ability to engineer exosomes to display proteins, incorporate specific nucleic acid and protein cargos, and target uptake by specific cells is rapidly growing. Exosomes could have great potential as tunable therapeutic delivery vehicles that are relatively easy to engineer, well-tolerated *in vivo* [39], and naturally efficient at mediating intracellular delivery of functional biomolecules. Translating exosome-based therapeutics to the clinic will require developing reproducible and economically viable methods for generating exosomes that are effective and well-tolerated *in vivo*. Essential to this effort is the systematic characterization of the effects of exosomal components on recipient cells. Similarly, it may also be necessary to generate methods for preventing the incorporation of undesirable producer cell-derived components into therapeutic exosomes. Although early efforts to engineer exosomes have demonstrated the promise of this approach, robust and general methods for incorporating therapeutic cargo molecules into exosomes and for targeting therapeutic exosomes to specific destinations *in vivo* are still required. Finally, the safety and efficiency of exosome-mediated delivery must be quantitatively benchmarked against existing gene delivery methods to identify key opportunities and challenges for harnessing and improving this approach. Each of these advances will enable exosome-mediated delivery of biomolecules to mature from an exciting scientific discovery to a viable therapeutic technology.
